# Investigation of Tinnitus Patients in Italy: Clinical and Audiological Characteristics

**DOI:** 10.1155/2010/265861

**Published:** 2010-06-23

**Authors:** Francesco Martines, Daniela Bentivegna, Fabiola Di Piazza, Enrico Martines, Vincenzo Sciacca, Gioacchino Martinciglio

**Affiliations:** ^1^Dipartimento di Neuroscienze Cliniche (DINeC), Sezione di Otorinolaringoiatria, Università degli Studi di Palermo, Via del Vespro, 129-90127 Palermo, Italy; ^2^Dipartimento di Biotecnologie Mediche e Medicina Legale (DIBiMed), Sezione di Audiologia, Università degli Studi di Palermo, Via del Vespro, 129-90127 Palermo, Italy; ^3^Dipartimento di Matematica e Applicazioni, Università degli Studi di Palermo, Via Archirafi, 34-90123 Palermo, Italy

## Abstract

*Objective*. 312 tinnitus sufferers were studied in order to analyze: the clinical characteristics of tinnitus; the presence of tinnitus-age correlation and tinnitus-hearing loss correlation; the impact of tinnitus on subjects' life and where possible the etiological/predisposing factors of tinnitus. *Results*. There is a slight predominance of males. The highest percentage of tinnitus results in the decades 61–70. Of the tinnitus sufferers, 197 (63.14%) have a hearing deficit (light hearing loss in 37.18% of cases). The hearing impairment results of sensorineural type in 74.62% and limited to the high frequencies in 58.50%. The tinnitus is referred as unilateral in 59.93%, a pure tone in 66.99% and 10 dB above the hearing threshold in 37.7%. It is limited to high frequencies in 72.10% of the patients with sensorineural hearing loss (SNHL) while the 88.37% of the patients with high-frequency SNHL have a high-pitched tinnitus (*χ* ^2^ = 66.26;*P* < .005). *Conclusion*. Hearing status and age represent the principal tinnitus related factors; there is a statistically significant association between high-pitched tinnitus and high-frequency SNHL. There is no significant correlation between tinnitus severity and tinnitus loudness confirming the possibility that neural connection involved in evoking tinnitus-related negative reactions are governed by conditioned reflexes.

## 1. Introduction

Tinnitus is an auditory phantom sensation, a “perception of a sound which results exclusively from activity within the nervous system without any corresponding mechanical, vibratory activity within the cochlea” with high prevalence rates in western societies [1–7].

Epidemiological studies showed that about one third of the population experiences tinnitus at least once in their life and about 1–5% develop serious psychosocial complications; in Italy an epidemiologic study based on questions to general population upon auditory dysfunctions evidenced a tinnitus prevalence percentage in 14.5% (8% in normal hearing subjects, 30.5% in presence of auditory dysfunctions) [8] while Girard et al., as reported by Deshaies et al., found a prevalence percentage of 5.2% in 41631 Canadians [9]. It was estimated that in Germany ~1.5 million, corresponding to 1.82% of population, have problems with tinnitus and 800.000, and 0.97% of the population, suffer so severely that they are in continuous medical treatment [10].

Persistent tinnitus may rapidly become a source of serious disturbance and handicap at psychological and socio-professional levels; in fact in 1–3% of the general population, the tinnitus affect the quality of life, involving sleep disturbance, work impairment, and psychiatric distress [11, 12].

The presence of tinnitus progressively increases with increasing age (12% after the age of 60; 5% in the 20–30 age group), and this is not so much correlated with senescence itself as with the frequent concomitant hearing loss. The tinnitus prevalence in fact increases to 70–85% of the hearing-impaired population [3–7, 13].

The tinnitus is much more common with a percentage >90%. There is no single pathophysiological pathway to explain the production of subjective tinnitus. All structures of the auditory system have been suggested as possible sites of generation for tinnitus, from periphery to auditory cortex [14, 15].

In most cases, despite appropriate medical examination, the origin of tinnitus is unknown but it is well documented in literature that tinnitus and hearing deficit are often related phenomena; either sensorineural hearing loss (SNHL) as well as noise-induced hearing loss are considered the first cause of tinnitus [16–18]. Tinnitus can be also due to other inner ear dysfunctions, such as those associated with sudden hearing loss or acoustic trauma, or part of otological and neurological diseases such as Ménière's disease, conductive hearing loss, acoustic neuroma or severe head injury.

Although tinnitus is commonly associated with hearing loss, other aetiological factors have emerged from the widest epidemiological studies of tinnitus prevalence and actually they were considered as potential causes of tinnitus and/or cofactors. As reported by Hoffman these factors include conditions such as vascular disease, diabetes, hypertension, autoimmune disorders, and degenerative neural disorders [16, 19, 20].

Even if in the past years there was a great consensus among the studies with respect to tinnitus sufferers, the knowledge on tinnitus onset and history, clinical presentation and audiological characteristics is still incomplete. Moreover it appears clear that there is an heterogeneity within the tinnitus population, the implication being that, as stated above, there may be a variety of aetiological mechanisms causing the tinnitus. Despite this, the doctor may not be in a situation where he can provide an aetiological link between the perception of tinnitus and one or more defined underlying pathologies. Finally, the perception of tinnitus may be linked to emotional changes, which may or may not be related to an organic pathology. All these unresolved questions underline the necessity of further investigations on tinnitus population.

The aim of this study was to focus on patients suffering from tinnitus who were referred to the Audiology Section of Palermo University in order to analyze both the clinical characteristics of tinnitus symptoms and, where possible, its etiological/predisposing factors; to evaluate both the tinnitus age correlation and the tinnitus hearing loss-correlation; to quantify its impact on subjects' life.

## 2. Materials and Methods

The study was conducted by the Audiology Section of the Department of Bio-technology of Palermo University on 312 subjects, 176 males and 136 females, ranging from 21 to 83 years of age, who suffered from tinnitus.

In this study all consecutive tinnitus patients were included except 6 subjects with coexisting psychotic disorders (2 subjects with schizophrenia and 4 subjects with mental retardation); these psychotic disorders presented the only exclusion criteria of this study because they did not permited the collaboration of patients.

After the personal data, all patients underwent to a careful general medical history to identify tinnitus-related pathologies and other health diseases; it followed an ENT specialist history and an otological examination. 

As for the analysis of data collected, the following parameters were considered: age, sex, hearing threshold, any audio vestibular-associated diseases, tinnitus laterality (unilateral or bilateral), tinnitus duration (acute: ≤3 months, subacute: 3–12 months, and chronic: >12 months), tinnitus measurements and subjective disturbance caused by tinnitus.

As for age, patients were divided into six age groups: from 21 to 30, 31 to 40, 41 to 50, 51 to 60, 61 to 70, and >70.

The audio/vestibular system was studied through threshold audiometry (considering the frequencies 0.5–1–2–3–4–8 kHz), tympanometry with stapedius reflex, auditory brain stem responses, nystagmography and Vestibular Evoked Miogenic Potentials. If necessary, in order to identify the cause of tinnitus, other specific exams were performed: in particular laboratory studies including hematocrit, blood chemistries, thyroid studies and lipid battery; Doppler ultrasonography (USG Doppler) evaluat blood flow disturbance in vertebral and basilar artery; middle/inner ear computer tomography study (CT) and, suspecting an acoustic neuroma or neurovascular conflict, a magnetic resonance imaging/Angio CT (MRI/Angio CT). 

Audiometric threshold was considered as the pure tone average for the frequencies 0.5–1–2–4–8 kHz and divided into: normal hearing (<20 dB); light hearing loss (21–40 dB); moderate hearing loss (41–70 dB); severe hearing loss (71–90 dB); profound hearing loss (>90 dB).

Hearing loss was also divided into three categories: conductive hearing loss; sensorineural hearing loss (high frequency including 3, 4 and 8 kHz; low frequency limited to 250–500 Hz; flat curve) and mixed loss.

The audiologic measurement of tinnitus included pitch matching and loudness matching. The pitch matching was determined asking to patient to compare tinnitus characteristics with the different tones proposed in head-phones and to choose the tone with frequency more similar to that of tinnitus; on the basis of the examined frequencies (0.125–0.25–0.5–1–2–4–8 KHz) it was able to identify three categories (Low-Middle-High pitched) considering undetermined overall cases of tinnitus with a frequency outside the range. The loudness matching was determined increasing, in head-phones, the magnitude of this tone for steps of 5 dB, until the patient declared to perceive with similar magnitude the tone and his tinnitus; this increase of magnitude of tone respect to hearing threshold was considered tinnitus loudness (0–5 dB, 5 dB, 10 dB, 15 dB, >15 dB above the hearing threshold).

To evaluate the perceived severity of tinnitus and its impact on life Tinnitus Handicap Inventory (THI - Newman e coll.) was administered. This tool is a 25-item survey that is composed of three subscales: a functional subscale (12 items), an emotional subscale (8 items) and a catastrophic response subscale (5 items) which address role and physical functioning, psychological distress, desperation and loss of control, respectively. Each item has 3 potential answers with “yes” assigned 4 points, “sometimes” 2 points, and “no” 0 points. This leads to a total score ranging from 0 indicating no tinnitus handicap and 100 the worst patients' annoyance. Classically it grades five categories of tinnitus severity: slight corresponding to a score 0–16; mild (18–36); moderate (38–56); severe (58–76); catastrophic (78–100) [21].

For the statistical analysis the *χ* ^2^ test, the Pearson's correlation coefficient (Pearson's *r*) and Anova test were calculated using the Matlab computer programme.

## 3. Results

**Figure 1:**
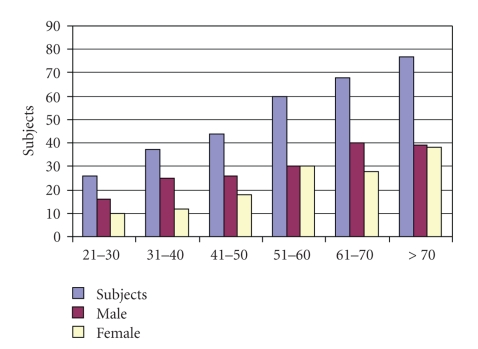
Distribution of the decades of age and sex (*χ* ^2^ = 5.15, df = 5, *P* = .39).

312 patients took part in the study, 176 (56.41%) males and 136 (43.59%) females, aged between 21 and 83 with a mean age of 58.37. The distribution of the decades of age and sex is indicated in Figure 1.

In 45.83% of cases, it was not possible to correlate the tinnitus with a known etiology; tinnitus was associated with chronic noise exposure in 27 cases (8.65%), acoustic trauma in 12 cases (3.85%), sudden hearing loss in 6 cases (1.92%), middle ear pathology in 35 cases (11.22%), degenerative cochlear disease as Menière and chemotherapeutic toxicity in 26 cases (8.33%). Moreover, it was diagnosed in 47 cases a coexisting Eustachian tube dysfunction and a temporomandibular Joint Syndrome in 5 cases (Table 1).

 As for other predisposing conditions for tinnitus, cardiovascular disease (hypertension, atherosclerosis or high blood viscosity) was identified in 87 patients; and metabolic disease was found in 23 cases.

The hearing loss accompanies tinnitus symptoms in 63.14% of patients; the hearing loss was identified as conductive hearing loss in 10.66% of cases, as sensorineural hearing loss in 74.62% of cases and mixed type in 14.72% of cases.

Of 147 patients with sensorineural hearing loss in 58.50% the hearing loss was limited to high frequencies; in the 11.56% to low frequencies; in 29.93% to a flat loss.

As for audiometric threshold, it was revealed: normal hearing in 36.86% of cases; light hearing loss in 37.18% of cases, moderate hearing loss in 14.1% of cases, severe hearing loss in 9.93% of cases and profound hearing loss in 1.92% of cases (Table 2). The mean values of audiometric threshold resulted are 30.94 ± 4.66 dB for the group with light hearing loss; 55.11 ± 6.69 dB for the group with moderate hearing loss; 79.98 ± 7.18 dB for the group with severe hearing loss and 97.5 ± 2.74 dB for the group with profound hearing loss. 

 Tinnitus was referred as unilateral in 187 patients (59.93%), 83 cases in the right ear and 104 cases in the left ear; bilateral in 74 patients (23.71%), and “in the head” in 51 cases (16.35%).

As for tinnitus duration, it was presented in acute form in 95 patients corresponding to 30.45%, subacute in 133 cases (42.63%) and chronic in 84 patients (26.92%).

In most cases, patients reported their tinnitus as a pure tone in 66.99% (209 cases); in relation to narrow-band in 27.88% (87 cases); at last, the tinnitus is not identifiable in 5.13% of cases (16 patients).

The tinnitus frequency, measured by the pitch matching test, was matched to high frequencies (4, 6, 8 KHz) in 172 patients (55.13%), to middle frequencies (1, 2, 3 KHz) in 56 patients (17.95%), to low frequencies (125, 250, 500 Hz) in 68 patients (21.79%) while it was variable and not identifiable in 16 patients (5.13%). Table 3 shows the tinnitus characteristics of the 312 subjects.

The 72.10% of the patients with sensorineural hearing loss had a high-pitched tinnitus while the 88.37% of the patients with high-frequency sensorineural hearing loss had a high-pitched tinnitus (Table 4).

**Figure 2:**
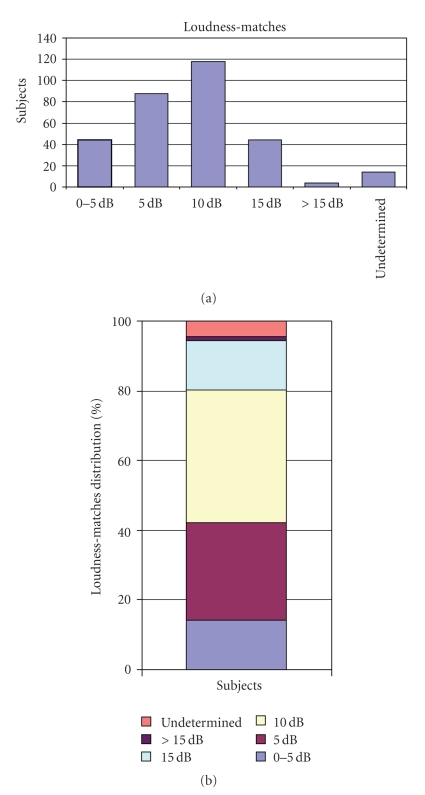
Distribution of the loudness matches: (a) absolute values; (b) relative values.

The loudness matches for tinnitus, as estimated by audiometry, was 0–5 dB above the hearing threshold in 14.1% of cases; 5 dB above the hearing threshold in 28.21% of cases; 10 dB above the hearing threshold in 37.8% of cases; 15 dB above the hearing threshold in 14.1% of cases; above 15 dB in 1.28% of cases; in 4.48% it was no able to identify the tinnitus loudness (Figure 2).

The tinnitus began suddenly in 41.02% (128 patients), increased in loudness overtime in 53.85% (168 cases), decreased in 8.33%, and was unchanged in 37.82% of participants (118 cases).

**Figure 3:**
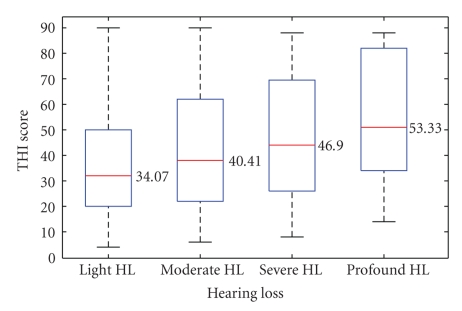
ANOVA test for THI score: Light HL 34.07 ± 20.09; Moderate HL 40.41 ± 22.95; Severe HL 46.90 ± 24.48; Profound HL 53.33 ± 30.11.

Regarding tinnitus annoyance and its impact on the quality of life, the THI identified five categories of tinnitus severity: slight grade was found in 59 patients (18.91%); mild grade in 102 patients (32.69%); moderate grade in 79 patients (25.32%); severe grade in 52 patients (16.67%) and catastrophic grade in 20 patients (6.41%). Figure 3 shows the Anova test for THI score and categories of hearing loss.

## 4. Discussion

Tinnitus is the general term for sound perception (roaring, hissing or ringing) that cannot be attributed to an external sound source. It has been reported that the condition affects from 5% to 32% of the world population, and in 25% of these cases, it is sufficiently severe to cause them to seek medical help [6, 8–10, 17, 18]. As it is confirmed in the present study in which the male prevalence is 56.41% (*P* = .39), usually there is a slight predominance of males respect to females; both Johansson and Arlinger (2003), Fabijanska et al. (1999) and Palmer et al. (2002) reported a minimally higher, but not statistically significant, prevalence overall for male than for female [17, 22, 23]. This could be correlated to high industrial noise exposure percentage among men more than females [24]. The epidemiological data have generally supported a strong association of tinnitus with increasing age; in particular the decade of age in which tinnitus affects more frequently is between 61 and 70 followed by lower decades (41–50) [4, 5, 10, 16, 22, 23]. Our study evidenced a highest percentage of tinnitus after the age of 70 followed by the decades 61–70 and 51–60; it is probably due both to the degree of hearing impairment and to the high incidence of predisposing conditions (i.e., cardiovascular and metabolic diseases) which, in tested population, it was progressively more evidenced in seniors.

Tinnitus is a multifactorial symptom, which can be induced by all types of hearing loss as well as by somatic and psychiatric disorders and pharmaceutical drugs. Axelsson (1991) described different diseases which were accompanied by tinnitus and the main causes leading to tinnitus were suggested. He was able to identify the aetiology of tinnitus in 81% of tinnitus tested cases [25]. In 26% of cases, Savastano (2008) was not able to correlate the tinnitus with a known etiology or anatomical site [26]. In our study the percentage of undetermined aetiology resulted 45.83%; this high value is due to a high number of chronic tinnitus sufferers examined in which it is more difficult to identify an aetiological factor also for the presence of coexisting psychological disorders associated to tinnitus.

Revised tinnitus literature seems to assume *de facto *a causal relationship between hearing loss and tinnitus but the value percentage of subjects with tinnitus associated with hearing loss is very variable ranging from 30% to 85% of cases [3–5, 9, 13]. This variability depends on the cohorts selected (i.e., age; professional noise-exposure; etc.) and it is strongly influenced by any condition such as middle-ear infections, cochlear hydrops, otosclerosis, antineoplastic drugs, that, leading to deterioration in hearing, are potential causes of tinnitus. In particular Steinmetz et al. (2009), Sindhusake et al. (2004), Kaharit et al. (2003) and Palmer et al. (2002) report a tinnitus/hearing loss correlation ranging from 12% to 50% in case of professional noise exposure [13, 20, 22, 27]. In this study, of a total number of 312 tinnitus affected, hearing loss was shown in 63.14% of cases with a high prevalence of sensorineural type (74.62%), supporting the actual theories that the loss of hair cell activity with consequent less effective functioning of the cochlea efferent system represents the commonest cause of tinnitus [14, 28].

When present, the sensorineural hearing loss was found, in most cases, limited to the high frequencies (58.50%) and of light-moderate degree (51.28%). These findings were also referred by Savastano, Satar et al. and Henry et al. [26, 29, 30]; also Nicolas-Puel et al. (2002) on 123 patients evidenced a prevalence high-frequency SNHL [24].

As for tinnitus characteristics it is accepted that there is a statistically significant association between high-pitched tinnitus and high-frequency SNHL; when subjects match their tinnitus pitch to a pure tone, most of the matches are at frequencies at which hearing is impaired [31–33]. The results of our study show a tinnitus as a pure tone in 66.99% and matched to high frequencies in 55.13%; moreover the 72.10% of the patients with SNHL had a high-pitched tinnitus while the 88.37% of the patients with high-frequency sensorineural hearing loss had a high-pitched tinnitus supporting data literature of a strong relationship, statistically significant, between the type of SNHL and the tinnitus-pitch (*χ* ^2^ = 66.1230, df = 6, *P* < .001).

More frequently, the subjective judgment of loudness matches for tinnitus was 10 dB above the hearing threshold (37.8%); this value suggests that most patients seek for specialist examination when the symptom is already disturbing [11, 12]. 

**Figure 4:**
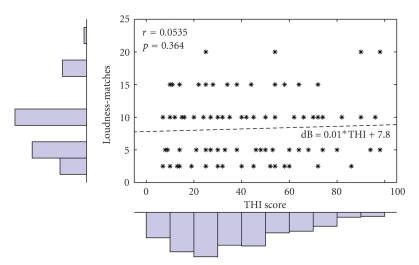
Regression analysis of THI and loudness matches.

The data work show that 23.08% of the subjects, 52 cases with severe grade and 20 cases with catastrophic grade according to THI test, have sleep disturbances and difficulty with any daily activity while no strong significant correlation was found between the magnitude of loudness matches for tinnitus measured by matching procedure and the tinnitus annoyance (*r* = 0.8712, *P* = .0544) (Table 5). The regression analysis confirms how stated above showing a correlation index of *r* = 0.053 with *P* = .364 (Figure 4). 

It may support the actual theory that the patient's reaction to tinnitus cannot be classified as a simple function of its psychoacoustic aspects but rather as a complex interaction between acoustic phantom symptoms, somatic attention and depressive symptoms.

## 5. Conclusions

Tinnitus is a distressing symptom, provoking an important decrease in the quality of life in 20% of tinnitus sufferers, that is strongly correlated to many factors. This paper, according to literature data, suggests that the hearing status and the old age represent the principal tinnitus-related factors; in most cases tinnitus is high-pitched and it is associated to high-frequency hearing loss with a significant statistical correlation between the two variables. The analysis of relationship between tinnitus severity (expressed as THI scores) and tinnitus loudness match clearly demonstrate lack of correlation between these variables. This finding support the possibility that neural connection involved in evoking tinnitus-related negative reactions is governed by conditioned reflexes.

## Figures and Tables

**Table 1:** Tinnitus etiologies and health factors associated.

Etiology	No. of cases	(%)
Chronic noise exposure	27	(8.65)
Acoustic trauma	12	(3.85)
Sudden hearing loss	6	(1.92)
Middle ear pathology	35	(11.22)
Otosclerosis	13	4.16
Chronic MO	12	3.84
Acute MO	10	3.20
Degenerative cochlear disease	26	(8.33)
Menière	17	5.44
Chemotherapeutic toxicity	9	2.88
Cerumen	3	(0.96)
Neurovascular cross compression with VIII cranial nerve	5	(1.60)
Retrocochlear pathology	3	(0.96)
Eustachian tube dysfunction	47	(15.06)
Temporomandibular Joint Syndrome	5	(1.60)
Unknown	143	(45.83)
Total	312	(100)

Other health factors associated with tinnitus	No. of cases	(%)

Cardiovascular diseases	87	(27.88)
Metabolic diseases	23	(7.37)

**Table 2:** Tinnitus participants: Audiological characteristics.

	Subjects	Total %	Sub group (%)
*Hearing of population*	312	100	
Normal Hearing	115	36.86	
Hearing Loss	197	63.14	

*Hearing threshold*	312	100	
Normal hearing	115	36.86	
Light HL	116	37.18	
Moderate HL	44	14.10	
Severe HL	31	9.93	
Profound HL	6	1.92	

*Hearing Loss*	197	63.14	(100)
Sensorineural HL	147	47.11	(74.62)
Conductive HL	21	6.73	(10.66)
Mixed HL	29	9.29	(14.72)

*Type of SNHL*	147	47.11	(100)
High frequencies SNHL	86	27.56	(58.50)
Low frequencies SNHL	17	5.45	(11.56)
Flat SNHL	44	14.10	(29.93)

**Table 3:** Tinnitus characteristics of the 312 subjects.

	Subjects No.	Total %
*Localization of tinnitus*		100
Right	83	26.61
Left	104	33.33
Bilateral	74	23.71
Within head	51	16.35

*Tinnitus duration*		100
Acute (≤3 months)	95	30.45
Subacute (3–12 months)	133	42.63
Chronic (>12 months)	84	26.92

*Subjective judgment of tinnitus type*		100
Pure tone	209	66.99
Narrow-band	87	27.88
Undetermined	16	5.13

*Tinnitus pitch*		100
High-pitched tinnitus	172	55.13
Middle-pitched tinnitus	56	17.95
Low-pitched tinnitus	68	21.79
Undetermined	16	5.13

*Type of tinnitus onset*		100
Suddenly	128	41.02
Slowly progressive	184	58.98

*Tinnitus matched loudness*		100
0–5 dB above the hearing threshold	44	14.1
5 dB above the hearing threshold	88	28.21
10 dB above the hearing threshold	118	37.8
15 dB above the hearing threshold	44	14.1
>15 dB above the hearing threshold	4	1.28
Undetermined	14	4.48

*Development of tinnitus loudness*		100
Has increased	168	53.85
Has decreased	26	8.33
Unchanged	118	37.82

*Degree of discomfort evaluated by THI*		100
Slight (I grade)	59	18.91
Mild (II grade)	102	32.69
Moderate (III grade)	79	25.32
Severe (IV grade)	52	16.67
Catastrophic (V grade)	20	6.41

**Table 4:** Tinnitus subjects and SNHL: distribution according to type of SNHL and tinnitus pitch.

Tinnitus pitch	Sensorineural hearing loss			Total
	High frequencies	Low frequencies	Flat	
	No.	No.	No.	No.
High-pitched	76	—	30	106
Middle-pitched	7	6	9	22
Low-pitched	2	10	5	17
Undetermined	1	1	—	2

Total	86	17	44	147

*χ* ^2^ = 66.1230, df = 6, *P* < .001.

**Table 5:** Distribution of 312 subjects according to tinnitus-matched loudness and Tinnitus Handicap Inventory.

THI	Tinnitus intensity						Total
(category)	0–5 dB	5 dB	10 dB	15 dB	>15 dB	undetermined	
	No.	No.	No.	No.	No.	No.	No.
Slight	12	17	21	8	—	1	59
Mild	14	32	38	14	1	3	102
Moderate	9	20	31	17	1	1	79
Severe	8	15	22	5	—	2	52
Catastrophic	1	4	6	—	2	7	20

Total	44	88	118	44	4	14	312

*χ* ^2^ = 70.06, df = 20, *P* < .001;

correlation index *r* = 0.8712, *P* = .0544.

## References

[1] Jastreboff PJ (1990). Phantom auditory perception (tinnitus): mechanisms of generation and perception. *Neuroscience Research*.

[2] Jastreboff PJ, Veron JA, Moller AR (1995). Tinnitus as a phantom perception: theories and clinical implications. *Mechanisms of Tinnitus*.

[3] Meikle M, Taylor-Walsh E (1984). Characteristics of tinnitus and related observations in over 1800 tinnitus clinic patients. *The Journal of Laryngology and Otology*.

[4] Axelsson A, Ringdahl A (1989). Tinnitus: a study of its prevalence and characteristics. *British Journal of Audiology*.

[5] Davis A, El Rafaie A, Tyler RS (2000). Epidemiology of tinnitus. *Tinnitus Handbook*.

[6] Heller AJ (2003). Classification and epidemiology of tinnitus. *Otolaryngologic Clinics of North America*.

[7] Adams PF, Hendershot GE, Marano MA (1999). Current estimates from the National Health Interview Survey, 1996. *Vital and Health Statistics. Series 10*.

[8] Quaranta A, Assennato G, Sallustio V (1996). Epidemiology of hearing problems among adults in Italy. *Scandinavian Audiology*.

[9] Deshaies P, Gonzales Z, Zenner HP, Plontke S, Paré L, Hébert S Quantification of the burden of disease for tinnitus caused by community noise. Background paper. http://www.chuq.qc.ca/oms/pdf/TinnitusBackgroundPaper2005.pdf.

[10] Pilgramm M, Rychlick R, Lebisch H, Siedentop H, Goebel G, Kirchhoff D Tinnitus in the Federal Republic of Germany: a representative epidemiological study.

[11] Dobie RA (2003). Depression and tinnitus. *Otolaryngologic Clinics of North America*.

[12] Andersson G (2003). Tinnitus loudness matchings in relation to annoyance and grading of severity. *Auris Nasus Larynx*.

[13] Steinmetz LG, Zeigelboim BS, Lacerda AB, Morata TC, Marques JM (2009). The characteristics of tinnitus in workers exposed to noise. *Brazilian Journal of Otorhinolaryngology*.

[14] Eggermont JJ (2003). Central tinnitus. *Auris Nasus Larynx*.

[15] Eggermont JJ, Roberts LE (2004). The neuroscience of tinnitus. *Trends in Neurosciences*.

[16] Hoffman HJ, Reed GW, Snow JB (2004). Epidemiology of tinnitus. *Tinnitus: Theory and Management*.

[17] Johansson MSK, Arlinger SD (2003). Prevalence of hearing impairment in a population in Sweden. *International Journal of Audiology*.

[18] Mercier V, Luy D, Hohmann BW (2003). The sound exposure of the audience at a music festival. *Noise and Health*.

[19] Sindhusake D, Golding M, Newall P, Rubin G, Jakobsen K, Mitchell P (2003). Risk factors for tinnitus in a population of older adults: the blue bountains hearing study. *Ear and Hearing*.

[20] Sindhusake D, Golding M, Wigney D, Newall P, Jakobsen K, Mitchell P (2004). Factors predicting severity of tinnitus: a population-based assessment. *Journal of the American Academy of Audiology*.

[21] Newman CW, Jacobson GP, Spitzer JB (1996). Development of the tinnitus handicap inventory. *Archives of Otolaryngology*.

[22] Palmer KT, Griffin MJ, Syddall HE, Davis A, Pannett B, Coggon D (2002). Occupational exposure to noise and the attributable burden of hearing difficulties in Great Britain. *Occupational and Environmental Medicine*.

[23] Fabijanska A, Rogowski M, Bartnik G, Skarzynski H, Hazell J Epidemiology of tinnitus and hyperacusis in Poland.

[24] Nicolas-Puel C, Faulconbridge RL, Guitton M, Puel J-L, Mondain M, Uziel A (2002). Characteristics of tinnitus and etiology of associated hearing loss: a study of 123 patients. *International Tinnitus Journal*.

[25] Axelsson A Causes of tinnitus.

[26] Savastano M (2008). Tinnitus with or without hearing loss: are its characteristics different?. *European Archives of Otorhinolaryngology*.

[27] Kaharit K, Zachau G, Eklof M, Sandsjo L, Moller C (2003). Assessment of hearing and hearing disorders in rock/jazz musicians. *International Journal of Audiology*.

[28] Noreña AJ, Eggermont JJ (2003). Changes in spontaneous neural activity immediately after an acoustic trauma: implications for neural correlates of tinnitus. *Hearing Research*.

[29] Satar B, Kapkin O, Ozkaptan Y (2003). Evaluation of cochlear function in patients with normal hearing and tinnitus: a distortion product otoacoustic emission study. *Kulak Burun Bogaz Ihtisas Dergisi*.

[30] Henry JA, Schechter MA, Regelein RT, Demis KC, Snow JB (2004). Veterans and tinnitus. *Tinnitus: Theory and Management*.

[31] Henry JA, Meikle M, Gilbert A, Hazell J Audiometric correlates of tinnitus pitch: insights from the Tinnitus Data Registry.

[32] Norena A, Micheyl C, Chéry-Croze S, Collet L (2002). Psychoacoustic characterization of the tinnitus spectrum: implications for the underlying mechanisms of tinnitus. *Audiology and Neuro-Otology*.

[33] König O, Schaette R, Kempter R, Gross M (2006). Course of hearing loss and occurrence of tinnitus. *Hearing Research*.

